# Mitochondrial H_2_S_n_-Mediated Anti-Inflammatory Theranostics

**DOI:** 10.1007/s40820-021-00689-1

**Published:** 2021-08-05

**Authors:** Won Young Kim, Miae Won, Seyoung Koo, Xingcai Zhang, Jong Seung Kim

**Affiliations:** 1 Department of Chemistry, Korea University, Seoul, 02841 Korea; 2 John A. Paulson School of Engineering and Applied Sciences, Harvard University, Cambridge, MA 02138 USA; 3 School of Engineering, Massachusetts Institute of Technology, Cambridge, MA 02139 USA

**Keywords:** Theranostics, Anti-inflammation, Mitochondria, Hydrogen polysulfides

## Abstract

The insistent demand for space-controllable delivery, which reduces the side effects of non-steroidal anti-inflammatory drugs (NSAIDs), has led to the development of a new theranostics-based approach for anti-inflammatory therapy. The current anti-inflammatory treatments can be improved by designing a drug delivery system responsive to the inflammatory site biomarker, hydrogen polysulfide (H_2_S_n_). Here, we report a novel theranostic agent **1** (**TA1**), consisting of three parts: H_2_S_n_-mediated triggering part, a two-photon fluorophore bearing mitochondria targeting unit (Rhodol-TPP), and anti-inflammatory COX inhibitor (indomethacin). In vitro experiments showed that **TA1** selectively reacts with H_2_S_n_ to concomitantly release both Rhodol-TPP and indomethacin. Confocal-microscopy imaging of inflammation-induced live cells suggested that **TA1** is localized in the mitochondria where the H_2_S_n_ is overexpressed. The **TA1** reacted with H_2_S_n_ in the endogenous and exogenous H_2_S_n_ environments and in lipopolysaccharide treated inflammatory cells. Moreover, **TA1** suppressed COX-2 level in the inflammatory-induced cells and prostaglandin E_2_ (PGE_2_) level in blood serum from inflammation-induced mouse models. In vivo experiments with inflammation-induced mouse models suggested that **TA1** exhibits inflammation-site-elective drug release followed by significant therapeutic effects, showing its function as a theranostic agent, capable of both anti-inflammatory therapy and precise diagnosis. Theranostic behavior of **TA1** is highly applicable in vivo model therapeutics for the inflammatory disease.

**Supplementary Information:**

The online version contains supplementary material available at 10.1007/s40820-021-00689-1.

## Highlights

- Theranostic agent **1** (**TA1**) was successfully developed to provide dual functions: anti-inflammatory therapy and precise diagnosis.
- **TA1** selectively reacts with H_2_S_n_ in the mitochondria in response to inflammatory reactions to simultaneously produce both indomethacin as a drug and ring-opened fluorescence-on Rhodol.
- Theranostic behavior of **TA1** was proven by in vitro and in vivo imaging.
- **TA1** reveals inflammatory site-selective drug release followed by significant therapeutic effects and is highly applicable in vivo model therapeutics for the inflammatory disease.

**Supplementary Information:**

The online version contains supplementary material available at 10.1007/s40820-021-00689-1.

## Graphical abstract

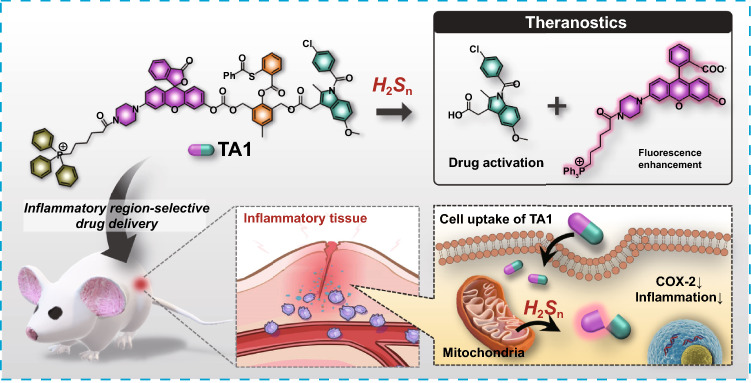

## Introduction

Inflammation is a biological response of body tissues to deleterious stimuli including injury, infection, damaged cells, pathogens, and irritants [1]. The immune system removes the injurious stimuli and initiates tissue repair through the inflammatory process [2]. Inflammation can be classified into two types: acute and chronic. Acute inflammation is an initial response to the harmful stimuli of the immune system, during which the movement of both plasma and white blood cells from the bloodstream to damaged tissues is increased, causing many biochemical events. This stage is accompanied by characteristic symptoms such as heat, pain, redness, swelling, and loss of tissue function in due course. Chronic inflammation, however, is long-term and leads to whole-body inflammation, producing steady and low-leveled inflammation throughout the body, which can develop from continuous acute inflammation [3]. This lasting chronic inflammation can eventually start markedly damaging tissues and organs, leading to the development of various chronic inflammatory diseases such as cardiovascular and bowel diseases, diabetes, arthritis, and cancer [4, 5]. Notably, it has been estimated that 15% of human cancers are associated with chronic inflammation [6].

Non-steroidal anti-inflammatory drugs (NSAIDs) exhibit excellent anti-inflammatory effects and are commonly prescribed medications to reduce pain, decrease fever, and prevent blood clots. NSAIDs reduce inflammation by inhibiting the activity of the cyclooxygenase enzymes (COX) involved in the synthesis of prostaglandins, which are key biological mediators in regulating inflammation [7]. Most NSAIDs are non-selective between COX-1 and COX-2. The NSAIDs inhibit COX-1 dependent generation of mucosal-protective gastric prostaglandins, thus considerably damaging the stomach and increasing the risk of gastrointestinal ulcers and bleeding, which are serious adverse effects of using NSAIDs. COX-2 selective inhibitors, on the other hand, exhibit lesser gastrointestinal side effects but cause thrombosis and substantially increase the risk of cardiovascular diseases [8]. These serious adverse effects are ascribed to the different roles and tissue localization of each COX isozyme. Due to these side effects of NSAIDs, there is an increasing demand for their selective delivery to specific inflammatory areas [9].

Hydrogen polysulfides (H_2_S_n_, *n* > 1) have drawn much attention because they are known as a fundamental regulator in biological redox-processes. Recent studies have suggested that the H_2_S_n_ molecules engage in various physiological functions such as cellular signal transduction, redox biology, and cytoprotective processes [10–12]. Endogenous H_2_S_n_ can be easily produced from the reaction of H_2_S with reactive oxygen species (ROS) [13, 14]. In the body, the enzymes, cystathionine γ-lyase (CSE) and cystathionine-β-synthase (CBS), are able to produce H_2_S, which can also generate persulfides that could, in turn, be converted into H_2_S_n_ [15]. The H_2_S_n_ thus generated is stored in mitochondria and can be released in response to physiological conditions such as oxidative stress and inflammation [16]. When inflammation occurs, the released H_2_S_n_ plays a crucial role in cytoprotection by scavenging ROS and also promoting nuclear localization of Nrf2 (Nuclear factor erythroid 2-related factor 2), which is one of the regulators that enhance anti-oxidant genes [17, 18].

**Scheme 1:**
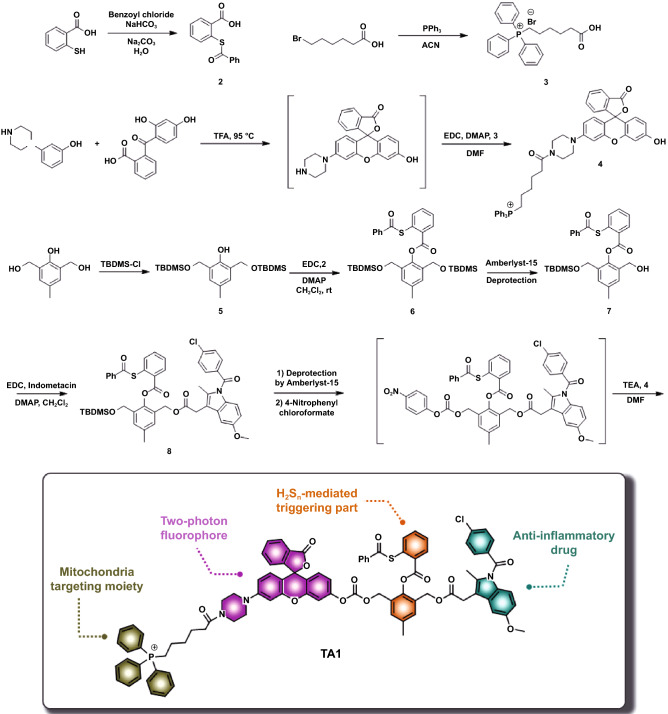
Synthetic routes of **TA1** and schematic illustration of H_2_S_n_-mediated anti-inflammatory theranostics system

As H_2_S_n_ is capable of triggering the selective delivery of anti-inflammatory drugs to inflammatory cells over normal cells, we for the first time designed H_2_S_n_-triggered anti-inflammatory theranostic agent **1** (**TA1**). “Theranostics” refers to the all-in-one system that integrates diagnostic and therapy, enabling precision and effective treatment. As seen in Scheme 1, the **TA1** consists of four crucial functional parts: aryl thioester as an exclusive H_2_S_n_ reactive site over other biological species [19]; triphenylphosphonium (TPP) salt unit as a mitochondrion targeting part, considering that H_2_S_n_ is mostly produced and stored in mitochondria; indomethacin as an anti-inflammatory drug able to inhibit COX enzyme that inhibits a synthesis of prostaglandins followed by reducing inflammation; and Rhodol dye as a fluorescent off–on reporter for the two-photon microscopy imaging. Such a theranostic system is a useful tool to visualize the interaction of prodrug with certain biomarkers and provides useful information to assess the therapeutic efficacy, such as localization and release kinetics of the drug in situ*.* [20–27]. Two-photon dye utilizes a long-wavelength light as an excitation source to offer several advantages, such as minimized tissue damage and deep tissue penetration, suitable for in vivo real-time bio-imaging [28–31].

Thus, in this system, the **TA1** can specifically localize in the inflammatory region, where the reaction of thioester moiety of the **TA1** with H_2_S_n_ selectively takes place in mitochondria, which subsequently triggers the self-immolation reaction to concomitantly release both indomethacin and Rhodol-TPP. The self-immolation reaction can promote the ring-opening of the Rhodol compartment, which emits a strong fluorescence (off–on) in the inflammatory site. Simultaneously, the released indomethacin can lessen the corresponding inflammation without exhibiting adverse effects on normal cells. Therefore, it is notable that the novel H_2_S_n_-mediated **TA1** could provide a promising dual function of anti-inflammatory treatment and precise diagnosis, simultaneously, at the inflammation site.

## Experimental

### Materials

Unless otherwise noted, all the materials for the synthesis were purchased from commercial suppliers (Sigma-Aldrich, Alfa, Samchun) and were used without further purification. All the reactions were carried out under the nitrogen. All procedures for work-up and purification were carried out with reagent-grade solvents under ambient atmosphere. Column chromatography was performed with silica gel 60 (Merck, 0.063 ~ 0.2 mm) as a stationary phase. Analytical thin layer chromatography was performed using Merk 60 F254 silica gel (pre-coated sheets, 0.25 mm thick). ^1^H and ^13^C NMR spectra were collected in NMR solvent (CDCl_3_) on a Bruker 500 MHz spectrometer. All chemical shifts are reported in ppm values using the peak of TMS as an internal reference. NMR data are reported as follows: chemical shifts, multiplicity (s: singlet, d: doublet, dd: doublet of doublets, t: triplet, q: quartet, m: multiplet, br: broad signal), and the coupling constant (Hz). The ESI-MS spectra were recorded using a Shimadzu LC/MS-2020 Series instrument and TSQ-LC-MS (Korea Basic Science Institute, Seoul).

### Spectroscopic Measurements

UV/Vis spectra were recorded on a Scinco S-3100 spectrometer, and fluorescence spectra were obtained using a Shimadzu RF-5301PC instrument. Stock solutions of **TA1** were prepared in DMSO. All excitation and emission slit widths were set at 5 nm/3 nm. The concentration of each of the samples was fixed at 10 μM in a total volume of 3 mL.

### HPLC Analysis

The aqueous solution of **TA1**, indomethacin, and Rhodol-TPP was prepared by mixing each compound (20 μM) in 10 mM PBS (pH 7.4). The reaction mixture of **TA1** with Na_2_S_3_ was prepared by mixing **TA1** (20 μM) and Na_2_S_3_ (200 μM) in 10 mM PBS (pH 7.4) containing cetrimonium bromide (200 μM; CTAB). Each sample was eluted at a flow rate of 1 mL/min using the mobile phase (Solvent A: deionized water containing 0.1% TFA, Solvent B: Methanol) with binary gradient (45–0% of Solvent A for 1 min, then 0% of Solvent A for 19 min, then 0–45% of Solvent A for 5 min). HPLC chromatogram was monitored using a 254 nm detector.

### Cell Culture

Murine macrophage cell lines (RAW 264.7) were purchased from the Korean Cell Line Bank. Cells were cultured in DMEM containing high glucose (Hyclone) with 10% fetal bovine serum (GIBCO) and 1% antibiotics (penicillin–streptomycin) (GIBCO). Cells were maintained in a humidified atmosphere with 5% CO_2_ at 37 °C.

### Cell Viability Assay

RAW 264.7 cells (2 × 10^4^ cells mL^−1^) were seeded on a 96 well microplate (SPL Life Science) and cells to obtain approximately 80% confluence in 24 h. After incubation, the cells were treated with **TA1** and control containing 1% DMSO for 24 h. To investigate the cell viability of the RAW 264.7 cells treated with probes, we conducted a CytoTox96® Non-Radioactive Cytotoxicity Assay Kit (Promega) according to the protocol. The Absorbance was measured at 490 nm by a SPECTRA MAX microplate reader (Molecular Devices). Cell viability assays were performed 3 times in triplicate, and the cell viability (%) was calculated as a percentage of measured absorbance compared to the control cells with 1% DMSO.

### One-photon Fluorescence Microscopy

RAW 264.7 Cells (5 × 10^5^ cells) were seeded on confocal glass-bottom dishes (SPL Life Science) and cells to obtain approximately 80% confluence in 24 h. After stabilization, the cells were treated with **TA1** (10 μM with 1% DMSO) for 2 h at 37 °C. To monitor the effect of the exogenous increase of H_2_S_n_, cells were pre-incubated with 5 µM Na_2_S_3_ for 2 h before **TA1** treatment. To monitor the effect of the endogenous increase of H_2_S_n_, cells were pre-incubated with 10 μg/mL LPS (Escherichia coli 055:B5, 10 μg kg^−1^, Sigma-Aldrich) for 16 h before **TA1** treatment. To monitor the effect of endogenous H_2_S_n_ inhibition, cells were pre-incubated with 1.0 mM PAG (DL-propargyl-glycine, Sigma-Aldrich, P7888) for 8 h before **TA1** treatment. After cells were washed with PBS, mitochondria was stained using a MitoTracker Red^FM^ (Invitrogen, USA) for 30 min. After incubation, cells were washed with PBS 3 times. One-photon fluorescence images were obtained by the probes with a confocal laser scanning microscope (Carl-Zeiss LSM 700). The fluorescence channel of **TA1** was excited at 488 nm, and the emission was collected by 500–600 nm.

### Two-photon Fluorescence Microscopy

RAW 264.7 Cells (5 × 10^5^ cells) were seeded on confocal glass-bottom dishes (SPL Life Science) and cells to obtain approximately 80% confluence in 24 h. After stabilization, the cells were treated with **TA1** (10 μM with 1% DMSO) for 2 h at 37 °C. The two-photon fluorescence microscopy images were obtained by exciting the probes with a spectral confocal microscope (Leica TCS SP2) with a mode-locked titanium-sapphire laser source (Coherent Chameleon) set at wavelength 800 nm in the focal plane in × 100 oil (NA = 1.30) objective lens. To obtain images at 500–600 nm range, internal PMTs were used to obtain the signals in an 8 bit unsigned 512 × 512 pixels at 400 Hz scan speed.

### Fluorescence Imaging of Acute Liver Injury in Mice Models

C57Bl/6 male mice (8 weeks old) were obtained from Orientbio (Seoul, Korea) and were established for the acute liver injury model using LPS. Animal care and experiments were conducted following guidelines by Korea University Institutional Animal Care Use Committee (KUIACUC No. 201900090). Mice were *i.v.* injected with a freshly prepared **TA1** solution (10 mg kg^−1^, in 5% DMSO) for 30 min. Then, 5% DMSO or LPS was intraperitoneally injected. At indicated time point, fluorescence images were collected at the indicated time point in vivo imaging machine (Maestro, CRi Inc., Woburn, MA, USA) with an emission filter from 550 to 750 nm (orange channel).

### ELISA Assay

Blood plasma was collected at the indicated time point, to conduct plasma enzymes and cytokines analysis in mice. Plasma activity of alanine aminotransferase (ALT) and aspartate aminotransferase (AST), as markers of hepatic damage; TNF-α and IL-1β, as markers of pro-inflammatory factors; PGE_2_, as a marker of COX-2 product at inflammatory sites were analyzed by an Enzyme-Linked Immunosorbent Assay Kit (Invitrogen.). Each enzyme activity signal was measured using a Multi-Detection Microplate Reader system (HIDEX), following the manufacturer’s procedure.

#### Statistical Analysis

The data of statistical significance represent the mean ± S.E. The data were analyzed by SAS 9.4 ver. Statistical significance was determined by a two-way ANOVA test with a Bonferroni test and Student’s t-test. Different letters statistically distinct signify data sets. (*p* < 0.05).

## Results and Discussion

### Synthesis of TA1

As shown in Scheme 1, the structure of target molecule **TA1** comprises three parts: 1) H_2_S_n_-reactive triggering part, 2) Rhodol-based two-photon fluorophore bearing mitochondria targeting TPP, and 3) anti-inflammatory drug, indomethacin. The **TA1** was synthesized by the following procedure. First, H_2_S_n_-reactive site **2** was prepared by s-acetylation of thiosalicylic acid using benzoyl chloride. The triggering unit, **6**, was synthesized via* tert*-butyldimethyl silyl (TBDMS) protection at both sides of aliphatic hydroxyl group of 2,6-bis(hydroxymethyl)-*p*-cresol, followed by 1-ethyl-3-(3-dimethylaminopropyl)carbodiimide (EDC) coupling reaction with **2**. Then, the mono-deprotection of the TBDMS group in **6** was conducted in the presence of amberlyst-15 to yield **7**. Compound **7** was then conjugated with indomethacin through EDC coupling to produce **8**. Finally, the other TBDMS group of **8** was further deprotected using amberlyst-15 and subsequently reacted with 4-nitrophenyl chloroformate for activation. The resulting intermediate was reacted with TPP-conjugated two-photon Rhodol fluorophore **4** (Rhodol-TPP) which was synthesized by EDC coupling of Rhodol and **3**, successfully yielding the desired product, **TA1** (Scheme 1). The detailed procedures of the synthesis and structurally characterized evidence for the compounds are provided in supporting information.

### Characterization of TA1

**Fig. 1:**
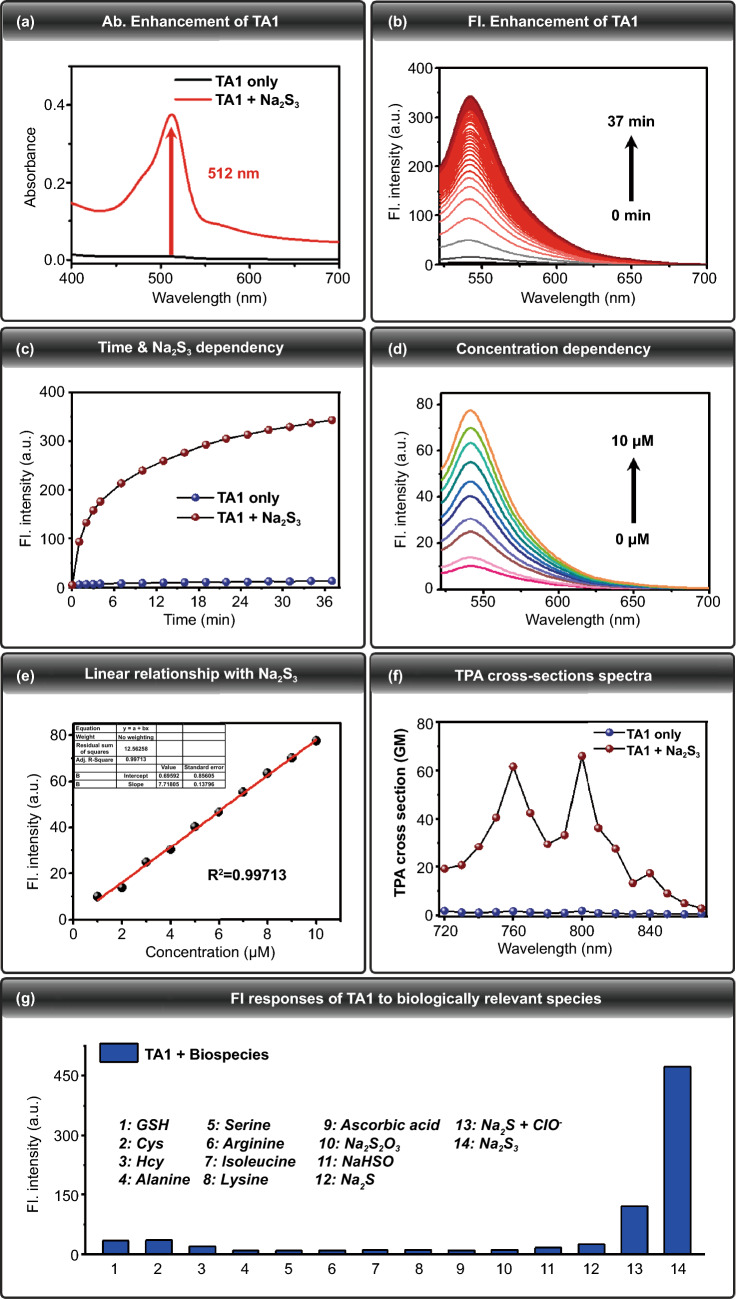
**a** Absorbance spectra of **TA1** (10 μM) in the absence of Na_2_S_3_ (black line), upon addition of Na_2_S_3_ (red line) (100 μM, 10 mM PBS buffer, 0.2% DMSO, 100 μM CTAB). **b** Time-dependent fluorescence spectral changes of **TA1** (10 μM) *λ*_ex_ = 512 nm, *λ*_em_ = 542 nm, upon addition of Na_2_S_3_ (100 μM, 10 mM PBS buffer, 0.2% DMSO, 100 μM CTAB). **c** Fluorescence intensity changes of **TA1** (10 μM) in the absence of Na_2_S_3_ (blue sphere), upon addition of Na_2_S_3_ (red sphere). **d** Fluorescence responses of **TA1** (10 μM) toward addition of a range of concentrations of Na_2_S_3_ (0 to 10 μM). **e** Linear relationship between the fluorescence intensity and Na_2_S_3_ concentrations. Data were acquired in 10 mM PBS buffer (0.2% DMSO, 100 μM CTAB) after incubation with Na_2_S_3_ for 2 min at 37 °C. *λ*_ex_ = 512 nm, *λ*_em_ = 542 nm. **f** Two-photon absorption (TPA) cross-sections spectra of **TA1** (10 μM) upon addition of Na_2_S_3_ (100 μM, 10 mM PBS buffer, 0.5% DMSO, 100 μM CTAB). **g** Fluorescence responses of **TA1** (10 μM) to biologically relevant species. Each bar represents fluorescence increases of **TA1** at 542 nm to 100 μM Na_2_S_3_ or other species. 1) 1 mM GSH; 2) 500 μM Cys; 3) 500 μM Hcy; 4) 100 μM Alanine; 5) 100 μM Serine; 6) 100 μM Arginine; 7) 100 μM Isoleucine; 8) 100 μM Lysine; 9) 100 μM Ascorbic acid; 10) 100 μM Na_2_S_2_O_3_; 11) 100 μM NaHSO_3_; 12) 100 μM Na_2_S; 13) 100 μM Na_2_S + 50 μM ClO^–^; 14) 100 μM Na_2_S_3_. The data were acquired in 10 mM PBS buffer (0.2% DMSO, 100 μM CTAB) after incubation for 30 min. *λ*_ex_ = 512 nm. (Color figure online)

**Fig. 2:**
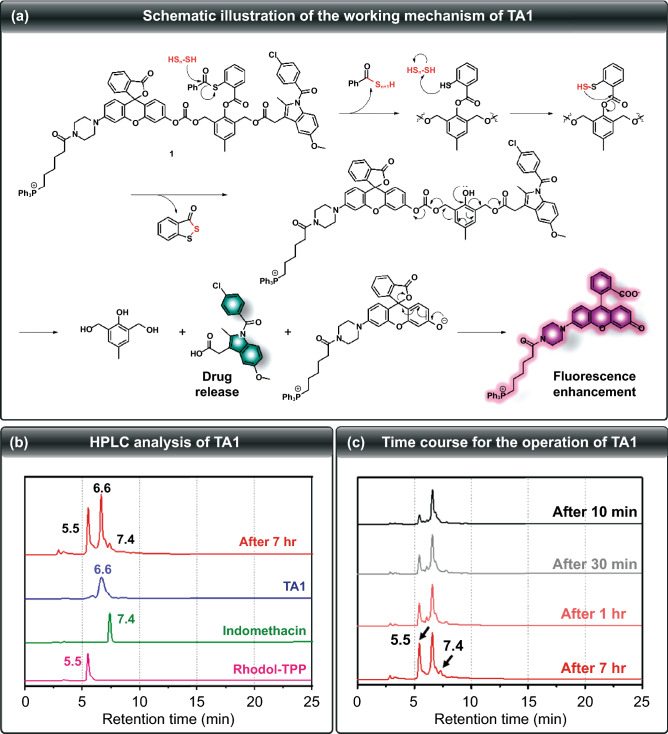
**a** Schematic illustration of the working mechanism of **TA1**. **b** HPLC chromatogram of **TA1** (blue line), indomethacin (green line), Rhodol-TPP (pink line), and the reaction mixture of **TA1** with Na_2_S_3 _(red line). **c** Time-dependent change of HPLC chromatogram of the reaction mixture of **TA1** with Na_2_S_3_. (COlor figure online)

To determine whether **TA1** could react with H_2_S_n_ to give an off–on fluorescence change, the photophysical change of **TA1** (10 μM) was investigated using UV–Vis absorption and fluorescence spectroscopy under simulated physiological conditions (10 mM PBS buffer, pH 7.4), in the presence and absence of Na_2_S_3_ (100 μM), H_2_S_n_ donor. UV–Vis absorption intensity of **TA1** (10 μM) markedly enhanced at 512 nm upon addition of Na_2_S_3_ (100 μM, 10 mM PBS buffer, 0.2% DMSO, 100 μM CTAB) (Fig. 1a). Time-dependent fluorescence intensity changes of **TA1** (10 μM) were observed, and they indicated a marginal fast (30 min) and gradual increase of its fluorescence intensity at 542 nm, upon treatment with Na_2_S_3_ (Fig. 1b, c). These results confirm that the **TA1** reacts with H_2_S_n_ and induces cleavage of H_2_S_n_-reactive triggering part to release the corresponding fluorophore, where the fluorescence off–on change is attributed to the ring-opening of Rhodol fluorophore upon self-immolation reaction (Fig. 2a). The fluorescence responses of **TA1** (10 μM) to H_2_S_n_ were also evaluated to investigate the sensitivity to different concentrations of Na_2_S_3_ (0 to 10 μM). As a function of Na_2_S_3_ concentration, gradually increasing fluorescence intensity of **TA1** (*λ*_ex_ = 512 nm) was observed, with a center at 542 nm (Fig. 1d). A linear correlation of various concentrations of Na_2_S_3_ with fluorescence intensities at 542 nm was also observed (Fig. 1e). Taken together, we conclude that **TA1** can effectively respond to H_2_S_n_ with reliable sensitivity under physiological conditions.

For two-photon confocal-microscopic imaging of the probe, TPA cross sections of **TA1** and **TA1** + H_2_S_n_ were initially investigated with rhodamine 6G as the reference molecule. Upon addition of Na_2_S_3_ (100 μM), **TA1** exhibited 66 GM with maximum TPA cross-section value at 800 nm under physiological conditions (PBS buffer, 0.5% DMSO, 100 μM CTAB), which validates that **TA1** can be sufficiently sensitized by two-photon absorption (Fig. 1f).

To determine the selectivity of **TA1** toward H_2_S_n_ over other biological species, fluorescence experiments with a series of other biologically relevant species were also performed. The **TA1** showed a high selectivity for Na_2_S_3_ over amino acids and other nucleophilic sulfur species such as glutathione (GSH), cysteine (Cys), homocysteine (Hcy), S_2_O_3_^2−^, HSO_3_^−^, and Na_2_S (Fig. 1g). Moreover, **TA1** was also found to be inert to other reductive species such as ascorbic acid. Upon treatment of a mixed solution of Na_2_S (200 μM) and ClO^−^ (50 μM) to generate H_2_S_n_ in situ, **TA1** showed a strongly enhanced fluorescence at 542 nm as well. These results conclude that **TA1** can selectively respond to H_2_S_n_ in the biological media containing various potential interferences.

### Proposed Mechanism of H_2_S_n_-responsive Activation of TA1

To verify that the proposed self-immolation cleavage mechanism of the theranostics system shown in Fig. 2a is reasonably operated, HPLC analysis of **TA1** in the presence of Na_2_S_3_ was undertaken. As shown in Fig. 2b, the retention time for **TA1**, Rhodol-TPP, and indomethacin was 6.6, 5.5, and 7.4 min, respectively. Time-course experiment upon reaction with H_2_S_n_ gave a Rhodol-TPP peak, cleaved from **TA1**, which gradually increased. Indomethacin release began after 1 h, indicating that **TA1** can release both Rhodol-TPP and indomethacin, simultaneously, upon reaction with H_2_S_n_ (Fig. 2c). Moreover, the ESI–MS spectrum of **TA1** in the presence of Na_2_S_3_ showed two peaks of *m/z* 380.12 and 759.30, corresponding to indomethacin and Rhodol-TPP, respectively (Fig. S13). This result supports the mode of action that **TA1** simultaneously releases both indomethacin and Rhodol-TPP in the presence of H_2_S_n_.

### Selective Activation of TA1 in vitro

**Fig. 3:**
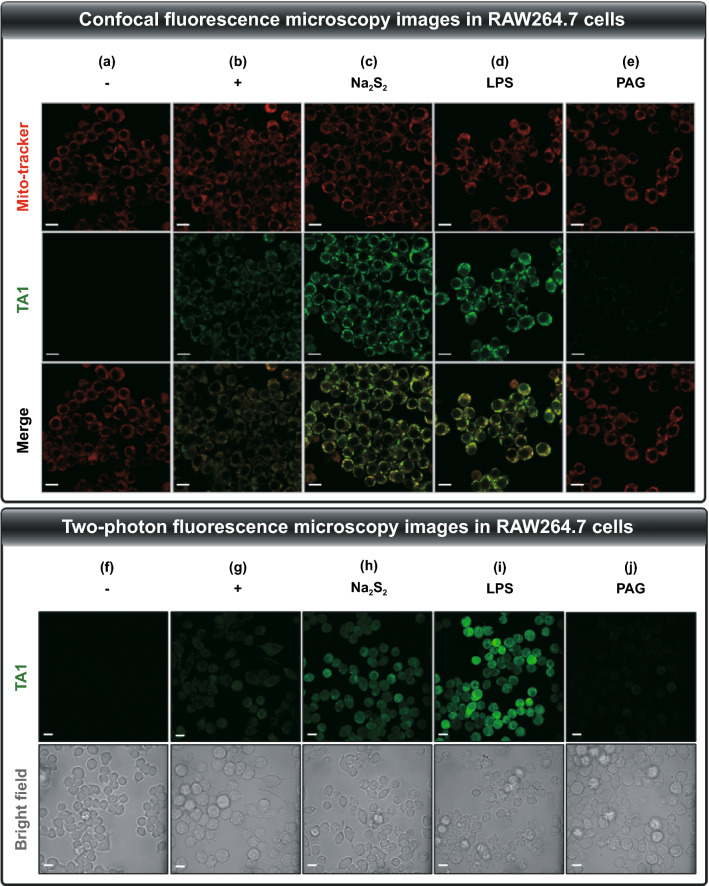
Confocal fluorescence microscopy images in RAW264.7 cells incubated with **TA1** (10 μM) for 2 h. The cells in each group were subjected to different treatments. **a** Control (1% DMSO), **b TA1**, **c TA1** + Na_2_S_2_ (5 μM), **d TA1** + LPS (1 μg mL^−1^), **e TA1** + PAG (1 mM). Fluorescence images of cells were collected at *λ*_ex_ = 488 nm, *λ*_em_ = 500–560 nm of **TA1** and at *λ*_ex_ = 579 nm, *λ*_em_ = 580–620 nm of Mito-tracker. Two-photon fluorescence microscopy images in RAW264.7 cells incubated with **TA1** (10 μM) for 2 h. The cells in each group were subjected to different treatments. **f** Control (1% DMSO), **g TA1**, **h TA1** + Na_2_S_2_ (5 μM), **i TA1** + LPS (1 μg mL^−1^), **j TA1** + PAG (1 mM). Fluorescence images of cells were collected at *λ*_ex_ = 800 nm, *λ*_em_ = 500–600 nm. Scale bar = 100 μm

The abovementioned results suggest that **TA1** selectively reacts with H_2_S_n_ and could be suitable for precise drug delivery as a potential theranostic agent. The **TA1** was then applied to mouse macrophage cell line, RAW264.7, as a bioassay model. First, from the LDH cytotoxicity assay, we found the low cytotoxicity of **TA1** in RAW264.7 cells, at various concentrations after 24 h-incubation (Fig. S14), thus suggesting that it could be further applied to an anti-inflammatory therapeutic system. Subsequently, confocal-microscopy images of the live RAW264.7 cells having endogenous H_2_S_n_ were obtained in the presence of **TA1** (10 μM) at 37 °C. The group treated with **TA1** exhibited brighter fluorescence as it responded to H_2_S_n_ compared to that exhibited by the control group (Fig. 3a, b and S15). The group further treated with exogenous Na_2_S_2_ (5 μM) displayed stronger fluorescence (Fig. 3c and S15). To further examine the responsiveness to endogenously produced H_2_S_n_ by perturbing the pool, RAW264.7 cells were pre-incubated with LPS (1 μg mL^−1^, 16 h), which can induce an inflammatory environment to trigger the overexpression of CSE mRNA, and thus promote the production of endogenous H_2_S_n_. Upon subsequent treatment of **TA1** (10 μM, 2 h), the cells displayed a remarkable increase in fluorescence intensity (Fig. 3d and S15).

On the contrary, the pretreatment of DL-propargylglycine (PAG, 1 mM; CSE inhibitor) significantly attenuated the fluorescence intensity of **TA1**, thus confirming that CSE contributed to the endogenous generation of H_2_S_n_ (Fig. 3e and S15). Moreover, mitochondrial localization of **TA1** was demonstrated via co-localization assays with Mito-tracker Red (Fig. 3b-e and S16), thereby proving the accessibility of **TA1** to mitochondrial H_2_S_n_. In addition, two-photon fluorescence microscopy images were collected from RAW264.7 cells for investigating the responsiveness of **TA1** toward H_2_S_n_ upon excitation at 800 nm (Fig. 3f-j and S15). These results are consistent with those from the one-photon fluorescence microscopy experiment. Collectively, the results thus indicate that **TA1** can react with endogenous cellular H_2_S_n_ in living cells, which can be directly visualized by fluorescence off–on changes with both one-photon and two-photon fluorescence microscopy.

### Anti-inhibitory Effects of TA1 in vivo

**Fig. 4:**
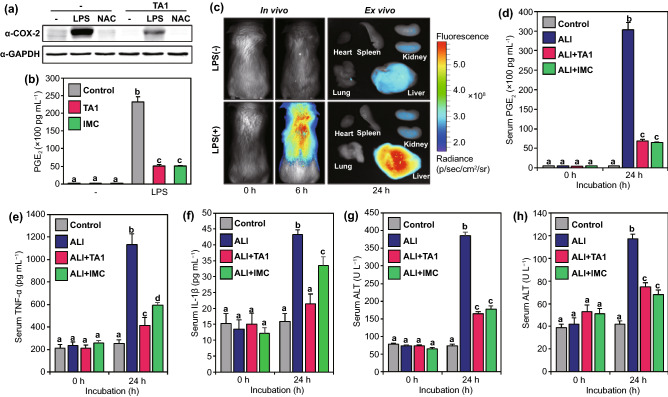
Anti-inflammatory effects of **TA1**. **a** Western blotting analysis of COX-2 protein in RAW264.7 cells. Cells were pre-incubated with LPS or NAC for 16 h before **TA1** treatment. **b** PGE_2_ levels of inflammation-induced RAW264.7 cells by LPS. **c** In vivo and ex vivo imaging of ALI mouse model. Mice were *i.v.* injected with **TA1** for 30 min and then treated with either 5% DMSO or LPS (10 μg kg^−1^). **d** Serum PGE_2_ levels of ALI mouse model. Levels of pro-inflammatory cytokines **e** TNF-α and **f** IL-1β on blood collected at indicated time points (0 and 24 h) after LPS (*i.p.*) administration. Levels of hepatic toxicity markers **g** AST and **h** ALT on blood collected at indicated time points (0 and 24 h) after LPS (*i.p*.) administration. Control (5% DMSO), ALI (LPS 10 μg kg^−1^), ALI + **TA1** (10 μg kg^−1^ LPS, 10 mg kg^−1^ **TA1**) and ALI + IMC (10 μg kg^−1^ LPS, 10 mg kg^−1^ IMC). Statistical significance was determined by a two-way ANOVA test with a post-hoc Bonferroni test. Different letters (*e.g.,* **a–d**) signify data sets that are statistically distinct (*p* < 0.05). ALI: LPS-induced acute liver injury, IMC: indomethacin, AST: aspartate transaminase, ALT: alanine aminotransferase

Following the results that **TA1** selectively responds to endogenous and exogenous H_2_S_n_ in living cells and shows diagnostic abilities, the therapeutic effect of **TA1** against inflammation was subsequently investigated by various biological tests. First, the western blot analysis of inflammation-induced RAW264.7 cells treated by LPS was implemented to explore COX-2 levels. The group treated with LPS exhibited high expression of COX-2 levels and another group treated with *N*-Acetyl cysteine (NAC, 1 mM for 12 h), a quencher of LPS-mediated inflammation, displayed decreased COX-2 levels (Fig. 4a). However, cells incubated with further treatment of **TA1** exhibited a significant reduction of COX-2 expression compared to that in the control group. This result signifies that **TA1** selectively releases indomethacin (IMC) upon reaction with H_2_S_n_, existing in the inflammatory environment, to reduce COX-2 levels. We also observed that PGE_2_ production increased in LPS-induced inflammatory response, whereas the levels were markedly reduced in **TA1** treated RAW264.7 cells (Fig. 4b). Moreover, an inflammation-induced mouse model was established to confirm the in vivo theranostic potential of **TA1**. Ahead of investigation, LPS was intraperitoneally injected in mice to cause hepatotoxicity and inflammation. Upon the injection of **TA1** into the LPS-induced acute liver injury (ALI) mouse model, the fluorescence expressions were observed to verify whether **TA1** is triggered at the inflammatory site. As seen in Fig. 4c and S17, the marked fluorescence enhancement of in vivo and ex vivo imaging was observed in the liver because **TA1** released both Rhodol-TPP and IMC upon reaction with H_2_S_n_ at the inflammatory site. Besides, the blood serum of the ALI mouse model was isolated to examine PGE_2_ level, which is representative of the level of inflammation. As shown in Fig. 4d, the PGE_2_ level in the ALI mouse model was significantly high, whereas it was reduced in the serum of the mice treated with either **TA1** or IMC. To investigate anti-inflammatory effects in the ALI mouse model, we further analyzed the production of pro-inflammatory cytokines, such as TNF-α and IL-1β in serum. The levels of TNF- α and IL-1β increased for 24 h in the ALI mouse model; however, the levels were significantly decreased in the group treated with **TA1** or IMC (Fig. 4e, f and S18). We also confirmed that **TA1** overcomes inflammatory responses, suppressing plasma levels of alanine aminotransferase (ALT) and aspartate aminotransferase (AST) in ALI mouse models (Fig. 4g, h). These results strongly suggest that the theranostic agent **TA1**, developed for the first time in this study, can selectively treat inflammation-related diseases by releasing the corresponding drug to the inflammatory site exclusively upon reaction with H_2_S_n_ and, simultaneously, aid in the diagnosis of the inflammation by fluorescence imaging in vivo.

## Conclusions

In summary, a novel H_2_S_n_ mediated anti-inflammatory theranostic agent, **TA1**, was developed to selectively deliver the NSAID, indomethacin, to the inflammatory region and to visualize the inflammation site using fluorescence off–on imaging of Rhodol-TPP by triggering H_2_S_n_. In this study, we found that **TA1** exerts a selectivity to H_2_S_n_ over other biological species such as amino acids and other reactive oxygen species, and the fluorescence signal of **TA1** is markedly enhanced not only in the endogenous and exogenous H_2_S_n_ environments but also in the inflammation-induced RAW264.7 cells by LPS. In addition, **TA1** exhibited the ability of two-photon excited fluorescence imaging, which is highly applicable to in vitro and in vivo biological experiments. Furthermore, we found that **TA1** could suppress both COX-2 level in the live cells and PGE_2_ level in blood serum, which are factors associated with inflammation-induced mouse models where H_2_S_n_ is overexpressed. Therefore, these results strongly suggest that **TA1,** we have first discovered has potential as a new theranostic agent as it is highly applicable to in vivo model therapeutics for inflammatory diseases.

## Supplementary Information

Below is the link to the electronic supplementary material.

Supplementary file1 (PDF 1370 kb)
