# Supplementary material for: Mitochondrial H2Sn-Mediated Anti-Inflammatory Theranostics
Source: Nanomicro Lett. 2021 Aug 5;13:168. doi: 10.1007/s40820-021-00689-1 (PMC8342730; doi:10.1007/s40820-021-00689-1)
Supplement: Supplementary file 1 — Supplementary file1 (PDF 1370 kb) [file 40820_2021_689_MOESM1_ESM.pdf]

Supporting Information for

Mitochondrial H_2S_n-Mediated Anti-Inflammatory Theranostics

Won Young Kim[1, †], Miae Won[1, †], Seyoung Koo[1, †], Xingcai Zhang[2, 3], Jong Seung Kim[1, *]

[1]Department of Chemistry, Korea University, Seoul 02841, Korea

[2]John A. Paulson School of Engineering and Applied Sciences, Harvard University, Cambridge, Massachusetts 02138, USA

[3]School of Engineering, Massachusetts Institute of Technology, Cambridge, Massachusetts 02139, USA

[†]Won Young Kim, Miae Won, and Seyoung Koo contributed equally to this wor.

*Corresponding author. E-mail: jongskim@korea.ac.kr (J. S. K.)

S1 Experimental

S1.1 Synthesis

Compound **2**: Thiosalicylic acid (1.5 g, 9.73 mmol, 1.0 eq.) was dissolved in a solution of sodium bicarbonate (2.45 g, 29.2 mmol, 3.0 eq.) and H_2O (30 mL). The solution was chilled to 0 °C by icebath and benzoyl chloride (1.13 mL, 9.73 mmol, 1.0 eq.) was added to the reaction mixture followed by further the addition of sodium carbonate (1.55 g, 14.6 mmol, 1.5 eq.). The reaction was maintained at 0 °C for 30 min, then allowed to increase to room temperature with stirring over two hours. Then, the addition of conc. aqueous HCl to acidify the mixture resulted in the formation of a white precipitant. The solid was collected by vacuum filtration and washed with H_2O to generate compound **2** as a pure white solid, yield 2.39 g, 95%. ^{1}H NMR (500 MHz, $CDCl_3$): δ 8.11 (dd, J = 7.8, 1.5 Hz, 1H), 8.03 (dd, J = 8.35, 1.2 Hz, 2H), 7.67 (dd, J = 7.75, 1.3Hz, 1H), 7.61 (dt, J = 7.45, 1.65 Hz, 2H), 7.53 (dt, J = 7.65, 1.4 Hz, 1H), 7.48 (t, J = 7.7 Hz, 2H).

Compound **3**: A mixture of 6-bromohexanoic acid (1.49 g, 7.62 mmol, 1.0 eq.) and triphenylphosphine (2.39 g, 9.15 mmol, 1.2 eq.) were dissolved in acetonitrile (10 mL). And the mixture solution was refluxed overnight. Then the reaction mixture was evaporated and subsequently washed with EtOAc. And it was allowed to be dried on vacuum afforded **3**, yield 3.27 g, 93%. ^{1}H NMR (500 MHz, $CDCl_3$): δ 7.80 (m, 7H), 7.77 (m, 2H), 7.72 (m, 6H), 3.62 (m, 2H), 2.36 (m, 2H), 1.67 (m, 6H).

Compound **4**: 2-(2,4-Dihydroxybenzoyl)benzoic acid (589 mg, 2.28 mmol, 1.0 eq.) and 1-(3-hydroxyphenyl)- piperazine (405 mg, 2.28 mmol, 1.0 eq.) were added to a pressure tube and dissolved in 15 mL of TFA. The reaction was stirred for 3 hours at 95 °C. After cooling, the reaction mixture was poured into 200 mL of ether. The resulting precipitate was collected, immediately redissolved in methanol, and then evaporated to dryness under reduced pressure to give a red solid. This crude fluorophore product (913 mg) was carried on without further purification. Specifically, it was dissolved in 5 mL anhydrous DMF and cooled to 0 °C. Compound **3** (1.25 g, 2.74 mmol, 1.2 eq.) and DMAP (557 mg, 4.56 mmol, 2.0 eq.) were added. A solution of EDC•HCl (875 mg, 4.56 mmol, 2.0 eq.) in 5 mL DMF was then added drop-wise. The reaction mixture was then stirred at room temperature overnight. The reaction mixture was concentrated in a vacuum. The crude product that resulted was purified by column

chromatography over silica gel using methanol (15%) in dichloromethane (DCM) as the eluent, afforded compound **4**, 416 mg, 22%, yield. ^{1}H NMR (500 MHz, $CDCl_3$): δ 7.98 (d, J = 7.6 Hz, 1H), 7.75 (m, 1H), 7.63 (t, J = 7.5 Hz, 1H), 7.15 (d, J = 7.5 Hz, 1H), 6.99 (m, 1H), 6.72 (m, 2H), 6.68 (m, 1H), 6.61 (m, 2H), 3.68 (m, 4H), 3.22 (m, 4H), 2.47 (m, 4H), 1.68 (m, 6H).

Compound **5** was synthesized according to the reported procedure [S1].

Compound **6**: Compound **2** (2.39 g, 9.26 mmol, 1.2 eq.) and compound **5** (3.06 g, 7.71 mmol, 1.0 eq.) were dissolved in dry DCM (30 mL). The solution was chilled to 0 °C by icebath and EDC•HCl (2.96 g, 15.4 mmol, 2.0 eq.) was added to the reaction mixture followed by further the addition of DMAP (1.88 g, 15.4 mmol, 2.0 eq.). The reaction was maintained at 0 °C for 30 min, then allowed to increase to room temperature with stirring overnight. After completion, the reaction mixture was diluted with DCM, and was washed 3 times with water. The organic layer was separated, dried over Na_2SO_4 and evaporated under reduced pressure. The crude product was purified by chromatography on silica gel (Hex/EtOAc 50:1) to give compound **6**, yield 3.77 g, 77%. ^{1}H NMR (500 MHz, $CDCl_3$): δ 8.33 (dd, J = 7.75, 1.55 Hz, 1H), 8.02 (dd, J= 8.4, 1.2 Hz, 2H), 7.75 (dd, J = 7.7, 1.3 Hz, 1H), 7.68 (dt, J = 7.5, 1.6 Hz, 1H), 7.61 (m, 2H), 7.47 (t, J = 7.7 Hz, 2H), 7.26 (s, 2H), 4.68 (s, 4H), 2.38 (s, 3H), 0.89 (s, 18H), 0.02 (s, 12H). ^{13}C NMR (125 MHz, $CDCl_3$): δ 189.11, 163.76, 142.31, 137.53, 136.56, 135.77, 133.66, 133.48, 133.02, 132.59, 131.64, 129.54, 129.18, 128.68, 127.57, 126.93, 60.29, 25.87, 21.30, 18.31, -5.40.

Compound **7**: Compound **6** (8.38 g, 13.2 mmol, 1.0 eq.) was dissolved in the solvent mixture (MeOH 10.0 mL and DCM 20.0 mL) and a portion of Amberlyst-15 was added to the solution. The reaction mixture was stirred for 2 h at room temperature and monitored by TLC (Hex/EtOAc 3:1). After completion, the solution was collected by vacuum filtration, and the crude product was purified by chromatography on silica gel (Hex/EtOAc 4:1) to give compound **7**, yield 3.35 g, 40%. ^{1}H NMR (500 MHz, $CDCl_3$): δ 8.31 (dd, J = 7.65, 1.65 Hz, 1H), 7.99 (dd, J = 8.3, 1.1 Hz, 2H), 7.71 (dd, J = 7.7, 1.4 Hz, 1H), 7.65 (dt, J = 7.4, 1.65 Hz, 1H), 7.61 (dd, J = 7.65, 1.5 Hz, 1H), 7.57 (dt, J = 7.6, 1.75 Hz, 1H), 7.44 (t, J = 7.55 Hz, 2H), 7.27 (s, 1H), 7.17 (s, 1H), 4.67 (s, 2H), 4.54 (s, 2H), 2.34 (s, 3H), 0.86 (s, 9H), -0.01 (s, 6H). ^{13}C NMR (125MHz, $CDCl_3$): δ 189.66, 164.76, 143.41, 137.48, 136.32, 133.88, 133.56, 133.45, 132.81, 131.73, 130.07, 129.93, 129.80, 129.07, 128.81, 128.44, 128.15, 127.62, 63.18, 60.46, 25.91, 21.11, 18.36, -5.34.

Compound **8**: Compound **7** (3.35 g, 5.27 mmol, 1.0 eq.) and indomethacin (2.26 g, 6.32 mmol, 1.2 eq.) were dissolved in dry DCM (30 mL). The solution was chilled to 0 °C by ice bath and EDC•HCl (2.02 g, 10.5 mmol, 2.0 eq.) was added to the reaction mixture followed by further the addition of DMAP (1.29 g, 10.532 mmol, 2.0 eq.). The reaction was maintained at 0 °C for 30 min, then allowed to increase to room temperature with stirring overnight. After completion, the reaction mixture was diluted with DCM, and was washed 3 times with water. The organic layer was separated, dried over Na_2SO_4 and evaporated under reduced pressure. The crude product was purified by chromatography on silica gel (Hex/EtOAc 4:1) to give compound **8**, yield 515 mg, 11%. ^{1}H NMR (500 MHz, $CDCl_3$): δ 8.23 (dd, J = 7.8, 1.3 Hz, 1H), 7.95 (dd, J = 8.4, 1.2 Hz, 2H), 7.72 (dd, J = 7.8, 1.1 Hz, 1H), 7.65 (dt, J = 7.75, 1.55 Hz, 1H), 7.61 (dt, J = 8.65, 2 Hz, 2H), 7.55 (m, 2H), 7.44 (dt, J = 8.65, 2 Hz, 2H), 7.40 (t, J = 7.6 Hz, 2H), 7.33 (s, 1H), 7.03 (s, 1H), 6.87 (d, J = 9.15 Hz, 1H), 6.85 (d, J = 2.5 Hz, 1H), 6.64 (dd, J = 8.95, 2.55 Hz, 1H), 5.11 (s, 2H), 4.63 (s, 2H), 3.68 (s, 3H), 3.56 (s, 2H), 2.28 (s, 3H), 2.22 (s, 3H), 0.87 (s, 9H), -0.01 (s, 6H). ^{13}C NMR (125 MHz, $CDCl_3$): δ 189.03, 170.44, 168.25, 163.81, 156.00, 143.73, 139.12, 137.71, 136.41, 136.06, 135.79, 134.11, 133.94, 133.68, 133.03, 132.77, 131.59, 131.12, 130.70, 130.57, 129.67, 129.35, 129.32, 129.11, 129.05, 128.67,

128.54, 127.54, 114.89, 112.48, 111.88, 101.09, 62.32, 60.00, 55.53, 30.11, 29.25, 25.85, 21.00, 13.27, -5.46.

TA1: Compound **8** (515 mg, 0.597 mmol, 1.0 eq.) was dissolved in the solvent mixture (MeOH 10 mL and DCM 20 mL) and a portion of Amberlyst-15 was added to the solution. The reaction mixture was stirred for 8 h at room temperature. After completion, the solution was collected by vacuum filtration, and the reaction mixture was concentrated in vacuum. To a solution of compound **8** in 5 mL of DCM, DIPEA (0.42 mL, 2.39 mmol, 4.0 eq.) was added at 0 °C. 4-nitrophenyl chloroformate (301 mg, 1.49 mmol, 2.5 eq.) in 5 mL DCM was then added. The reaction mixture was stirred at room temperature for 4 h. And the reaction mixture was concentrated under reduced pressure. The resulting reaction mass was diluted with 5 mL anhydrous DMF. TEA (0.33 mL, 2.39 mmol, 4.0 eq.) was added, followed by addition of compound **4** (416 mg, 0.496 mmol, 0.83 eq.) as a solution in DMF (5 mL) at 0 °C. The reaction mixture was stirred at room temperature for overnight. After completion, the reaction mixture was diluted with DCM, and was washed 3 times with water. The organic layer was separated, dried over Na_2SO_4 and evaporated under reduced pressure. And the crude product was purified *via* silica gel column chromatography using methanol (10.0%) in DCM as the eluent to give **TA1**, 34 mg, 4.2% yield. ^{1}H NMR (500 MHz, $CDCl_3$): δ 8.23 (d, J = 9.15 Hz, 1H), 8.19 (m, 4H), 8.02 (d, J = 7.55 Hz, 2H), 7.77 (m, 15H), 7.52 (m, 4H), 7.20 (m, 4H), 6.89 (m, 2H), 6.84 (m, 2H), 6.72 (m, 2H), 6.65 (m, 5H), 3.91 (m, 2H), 3.85 (m, 2H), 3.83 (m, 3H), 3.70 (m, 4H), 3.61 (m, 2H), 3.24 (m, 4H), 2.63 (m, 3H), 2.43 (m, 4H), 2.27 (m, 3H), 1.67 (m, 6H). ^{13}C NMR (125 MHz, $CDCl_3$): δ 174.0, 172.0, 171.2, 169.4, 169.3, 164.7, 155.5, 153.0, 152.6, 152.3, 152.2, 152.1, 151.7, 145.2, 135.1, 135.0, 135.0, 133.9, 133.8, 133.7, 133.6, 132.0, 131.8, 131.2, 130.5, 130.4, 130.3, 129.8, 129.2, 129.1, 128.8, 128.7, 127.4, 126.6, 125.1, 125.0, 124.4, 124.1, 122.6, 118.7, 118.0, 117.4, 117.0, 112.3, 110.5, 109.2, 102.3, 82.8, 55.8, 54.1, 53.5, 51.5, 48.5, 48.0, 45.2, 42.4, 41.1, 33.8, 33.4, 32.8, 30.0, 29.9, 29.8, 29.7, 29.6, 29.5, 24.7, 24.4, 24.2, 22.8, 22.6, 22.5, 22.4, 18.7, 17.4, 13.5, 12.1. ESI-MS calc. for $[C_{91}H_{76}ClN_3O_{14}PS]^+$ 1532.45, found 1532.10.

S1.2 Western Blotting

Protein expression levels of COX-2 were determined by western blotting analysis. Cells were pre-incubated with LPS or NAC for 16 h and cells were then treated with control as a 1% DMSO, 10 µM **TA1** for 24 h. Briefly, attached cells were washed with ice-cold PBS solution three times and scraped to collect cell pellets. After removal of the PBS, a radioimmunoprecipitation assay (RIPA) lysis buffer containing protease inhibitors provided by the manufacturer (Up-State) was added into the cell pellets to obtain protein lysates. A BCA assay was conducted to measure the protein concentration of each cell line and then protein (30 µg/lane) from each cell line was loaded onto a sodium dodecyl sulfate polyacrylamide gel electrophoresis (SDS-PAGE) for separating the proteins into bands. The separated protein bands were transferred to polyvinylidene difluoride (PVDF) membranes (Merck Millipore) and these membranes were incubated with COX-2 antibodies (Cell Signaling Technology) at 1/1000, GAPDH antibodies (Santa Cruz Biotechnology) at 1/3000, or dilution overnight at 4 °C. The resulting membranes were washed Tris-buffered saline with tween-20 (TBS-T) and then incubated with the anti-rabbit horseradish peroxidase (HRP)-conjugated secondary antibodies (Santa Cruz Biotechnology) for 12 h at 4 °C. To detect immunoreactive protein bands, enhanced chemiluminescence reagents (Luminate, Merk Millipore) were used according to the manufacturer’s instructions.

S2 Supplementary Figures

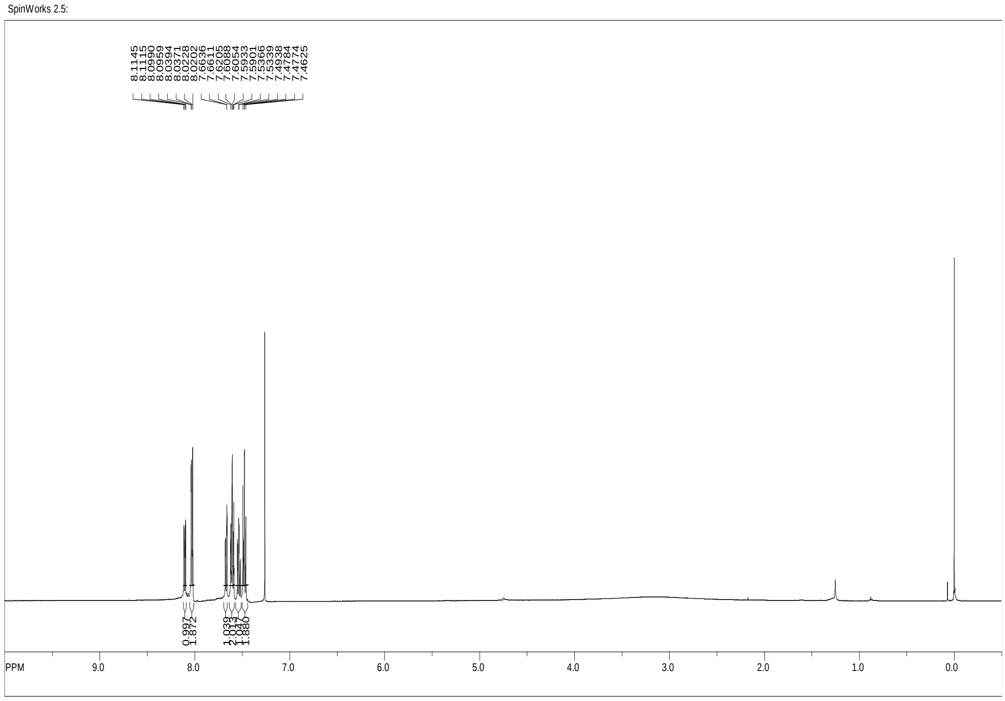

Fig. S1 ^{1}H NMR spectrum (500 MHz) of **2** in $CDCl_3$

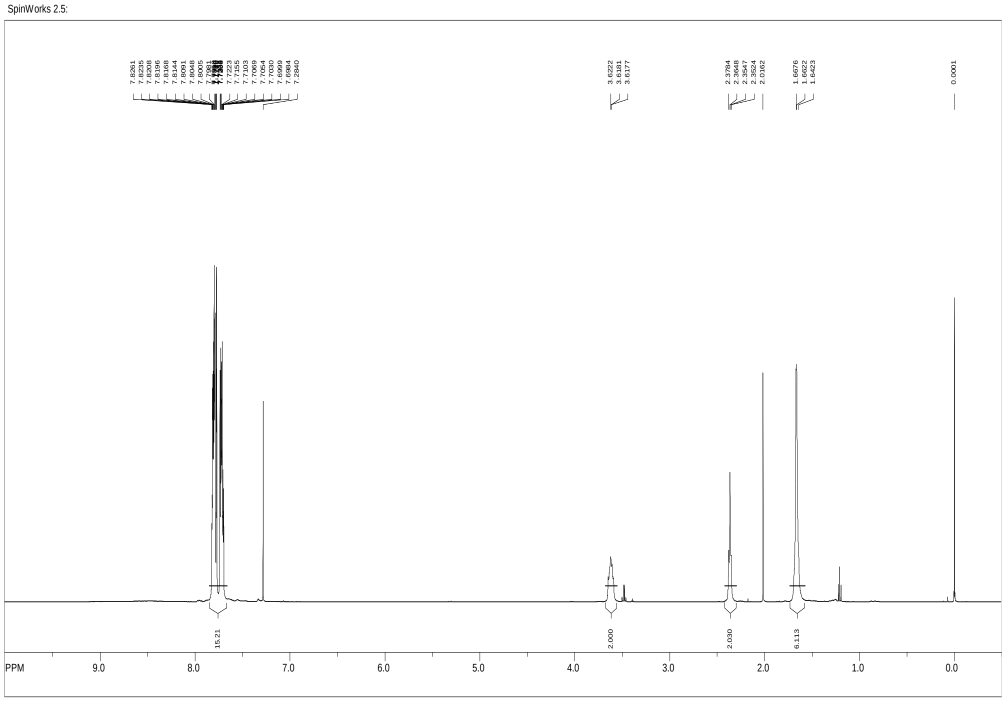

Fig. S2 ^{1}H NMR spectrum (500 MHz) of 3 in $CDCl_3$

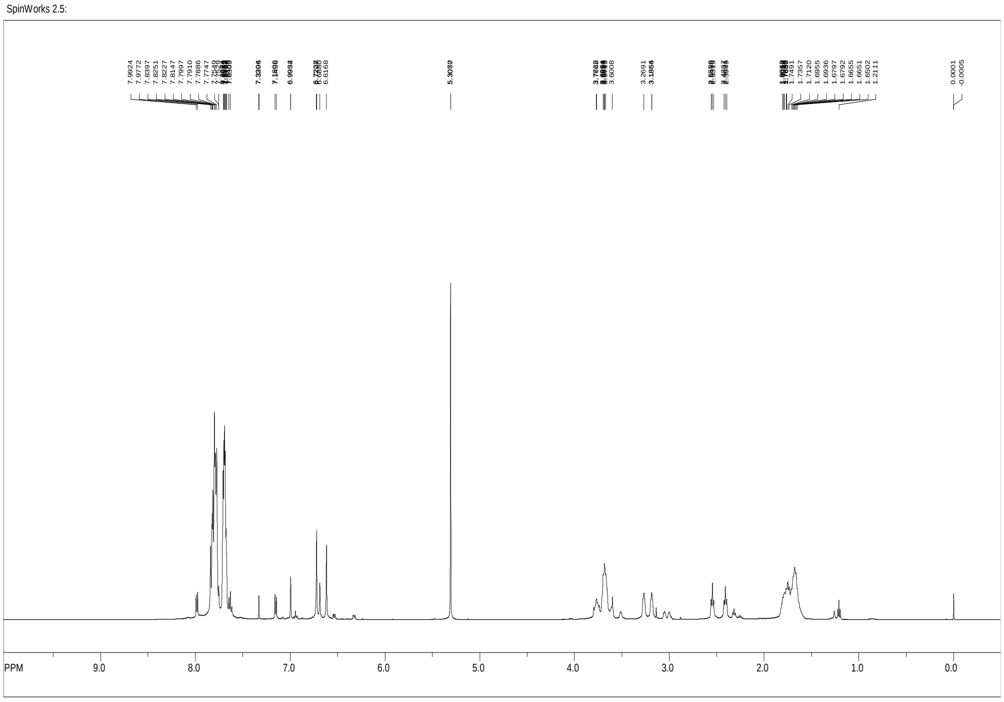

Fig. S3 ^{1}H NMR spectrum (500 MHz) of **4** in $CDCl_3$

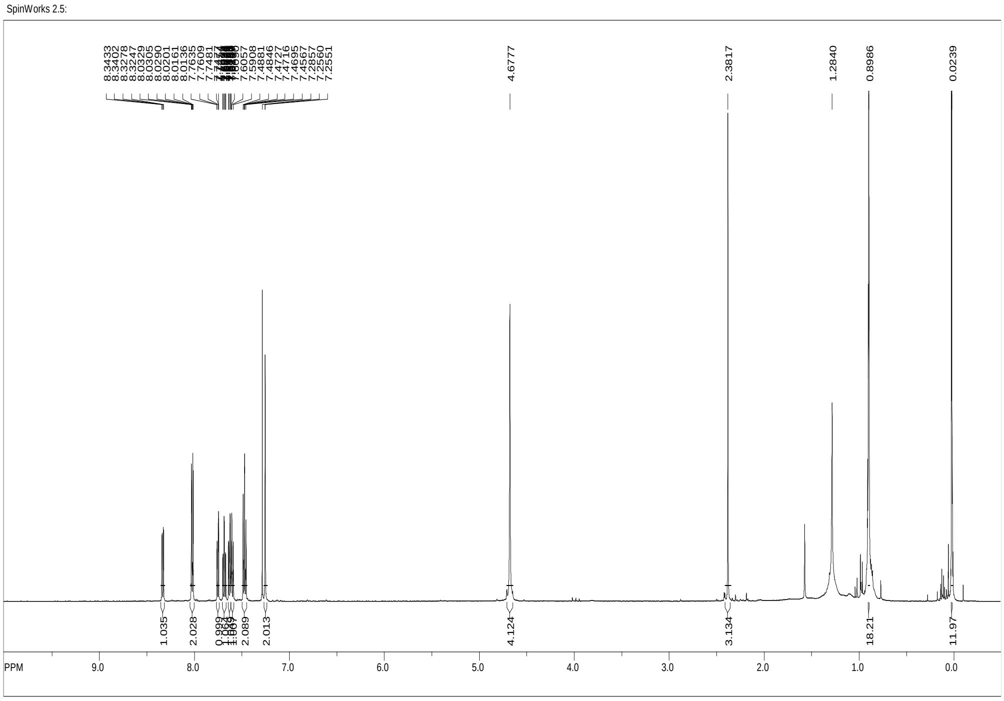

Fig. S4 ^{1}H NMR spectrum (500 MHz) of **6** in $CDCl_3$

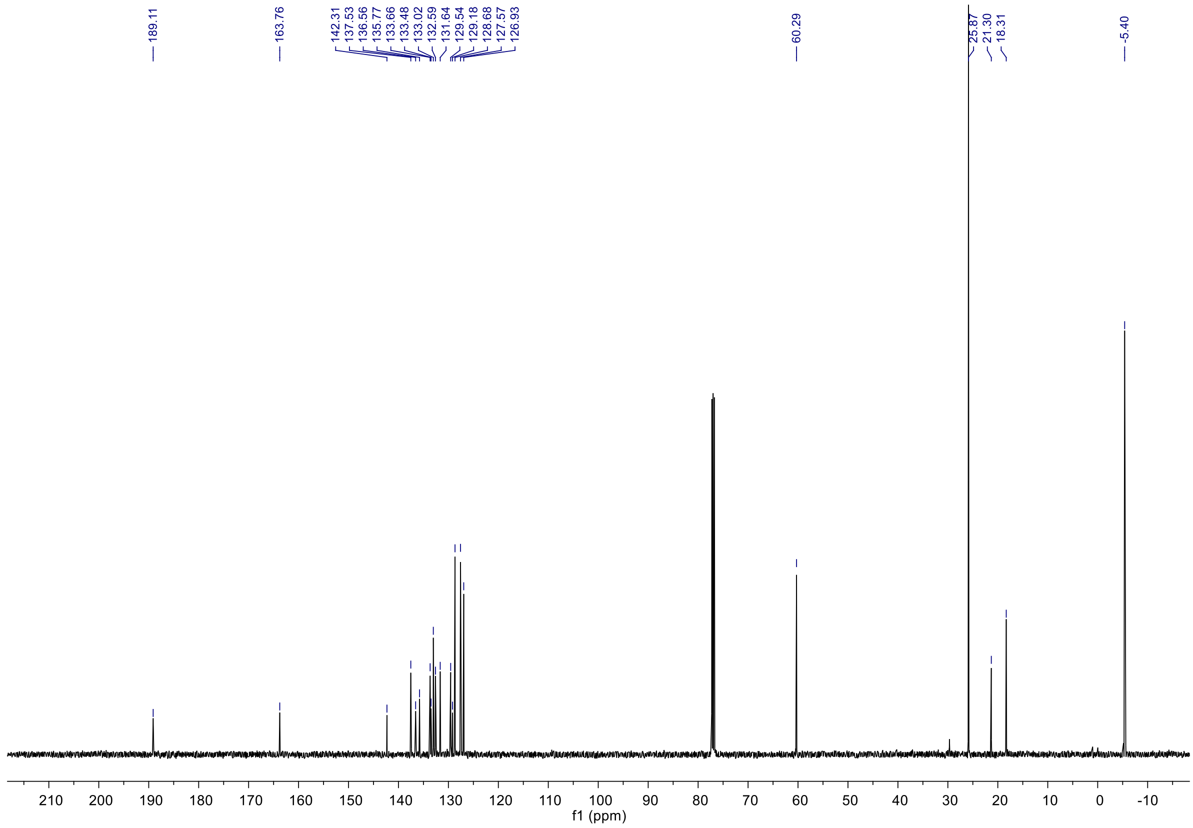

Fig. S5 ^{13}C NMR spectrum (125 MHz) of **6** in $CDCl_3$

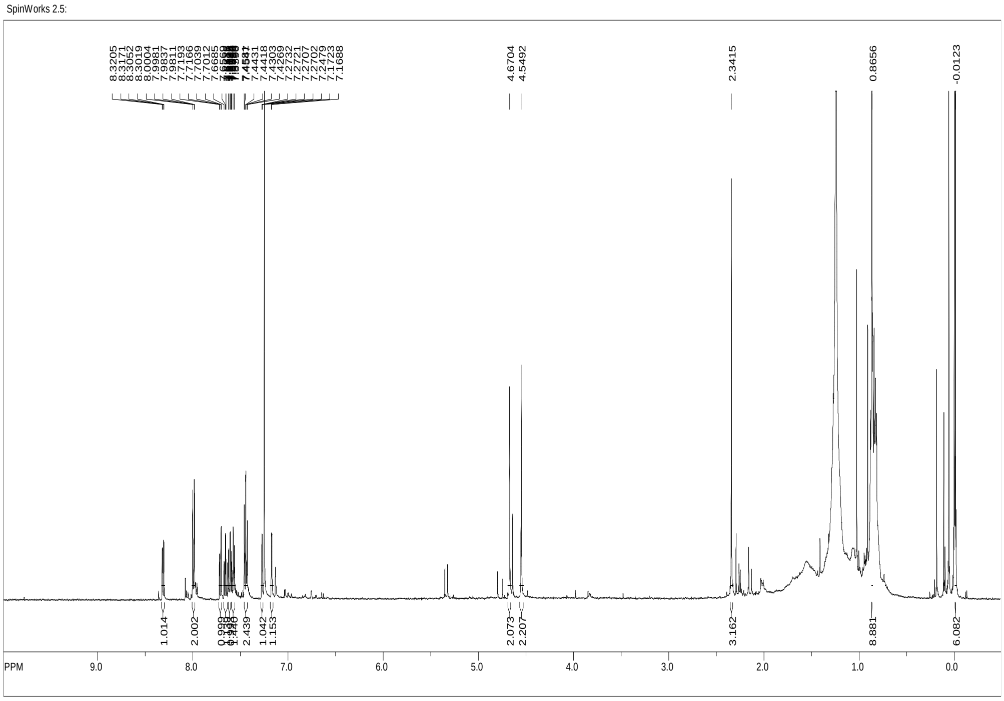

Fig. S6 ^{1}H NMR spectrum (500 MHz) of **7** in $CDCl_3$

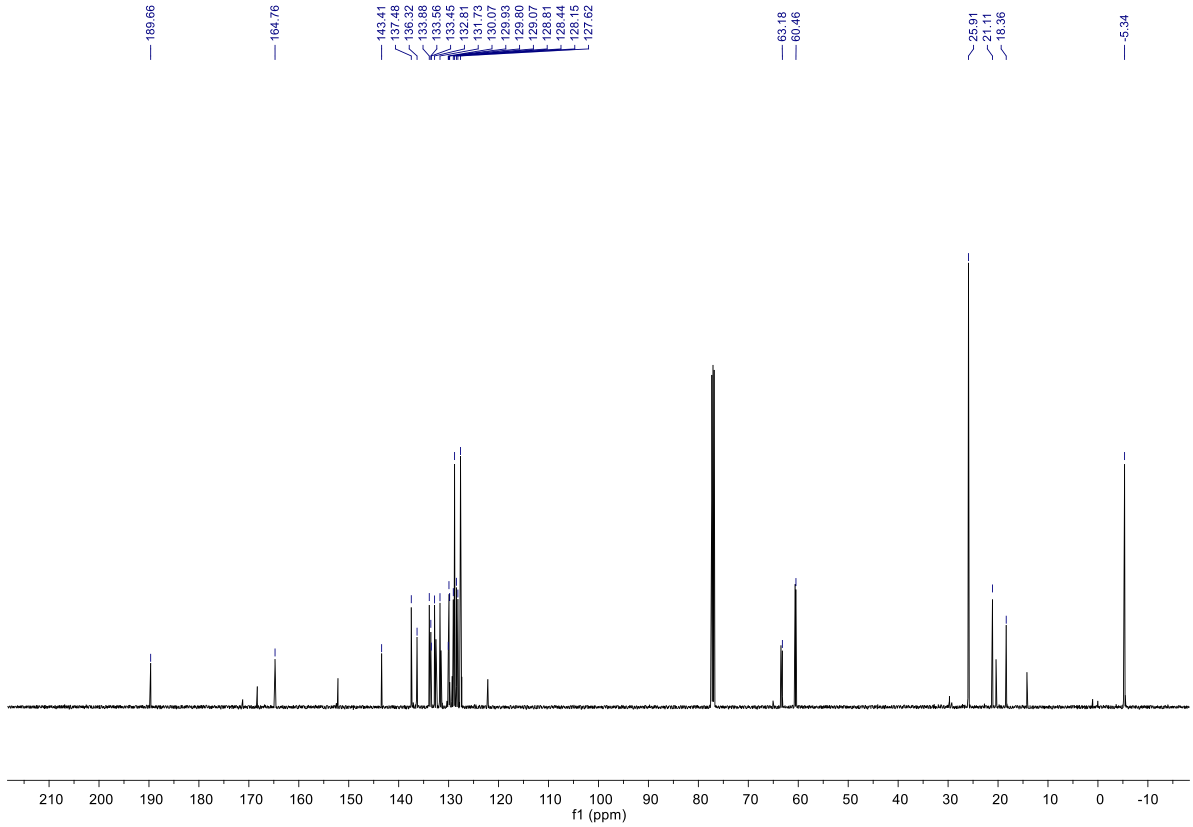

Fig. S7 ^{13}C NMR spectrum (125 MHz) of **7** in $CDCl_3$

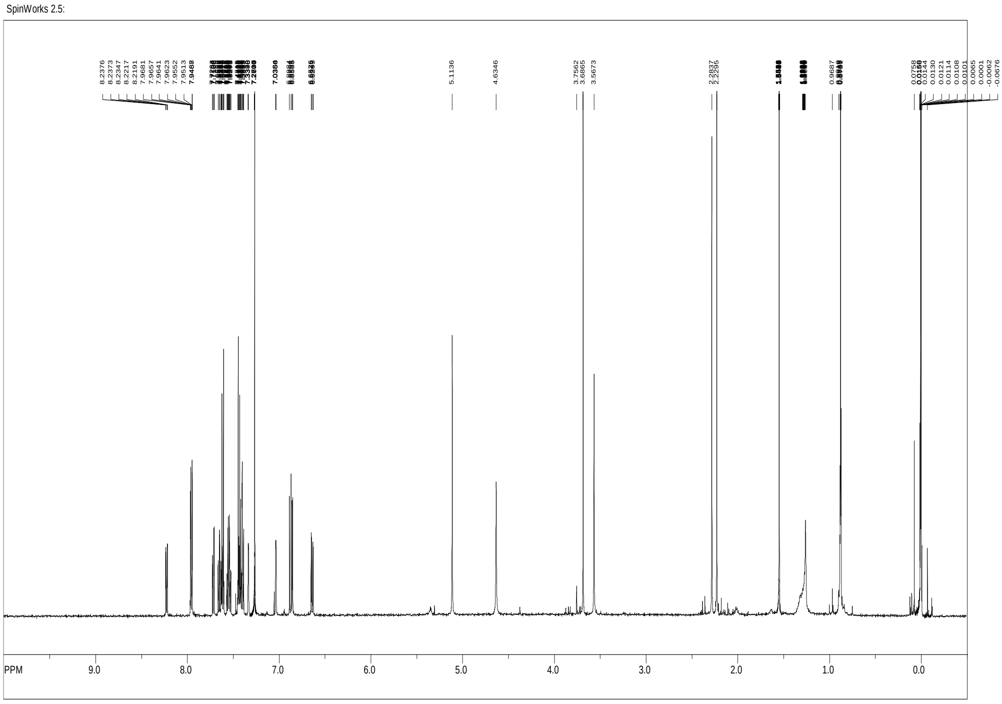

Fig. S8 ^{1}H NMR spectrum (500 MHz) of **8** in $CDCl_3$.

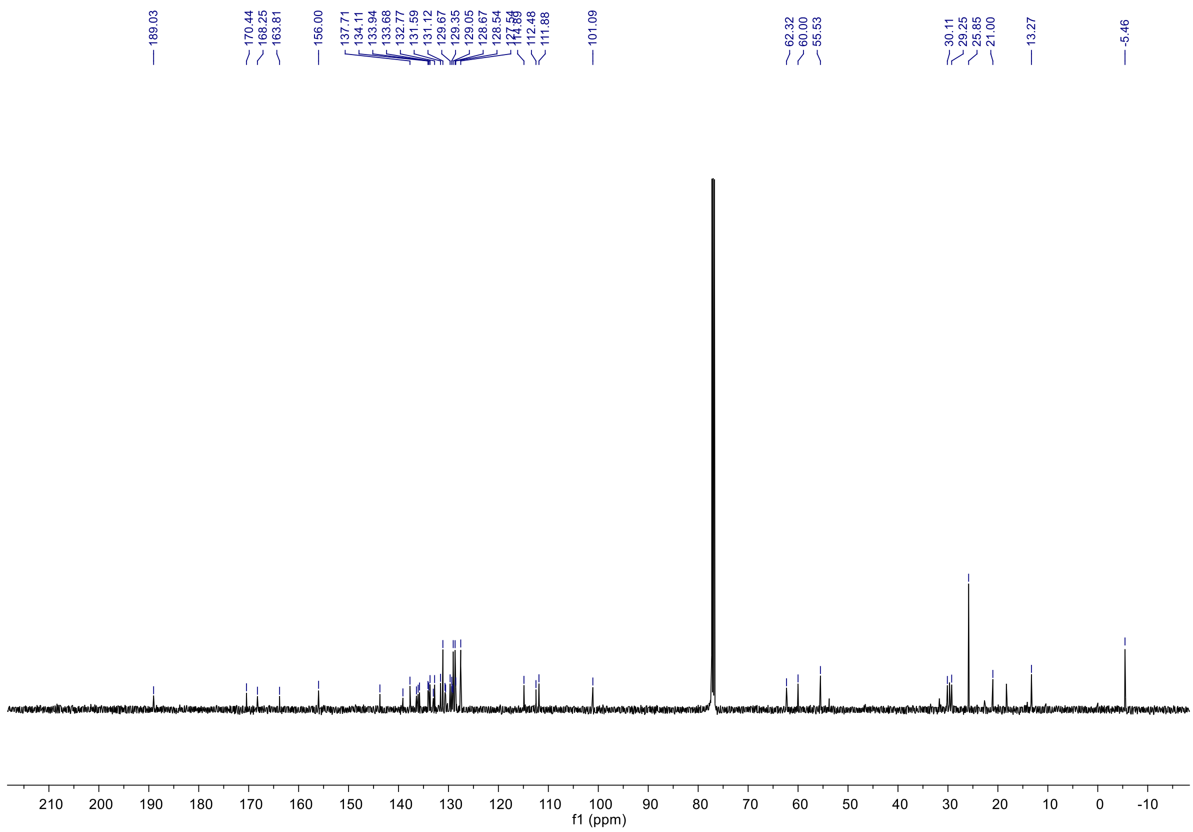

Fig. S9 ^{13}C NMR spectrum (125 MHz) of **8** in $CDCl_3$

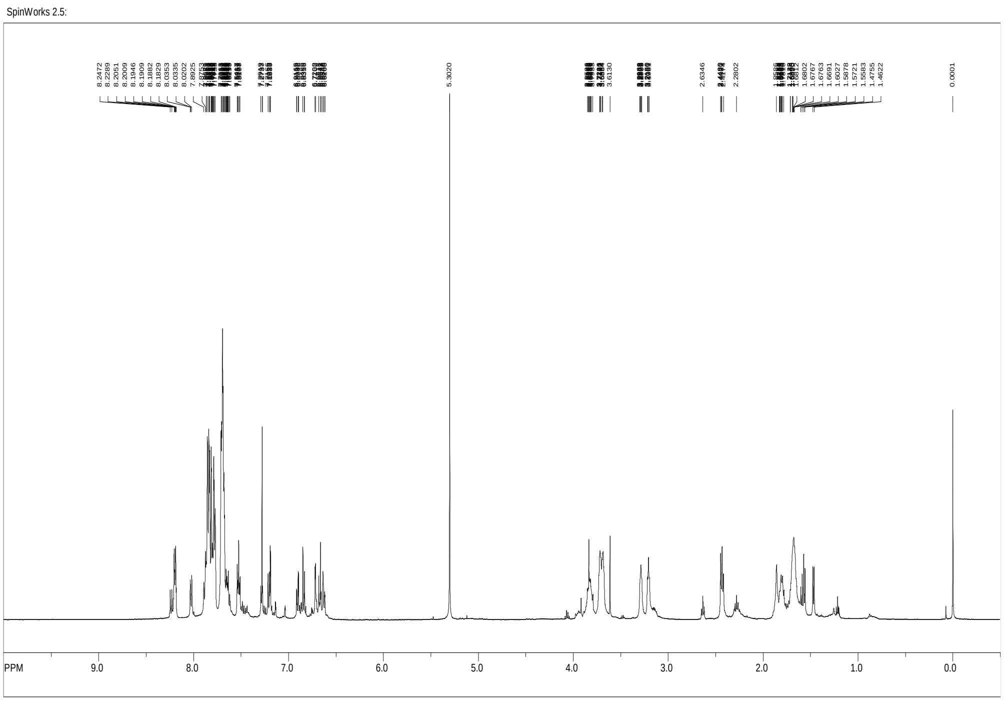

Fig. S10 ^{1}H NMR spectrum (500 MHz) of **TA1** in $CDCl_3$

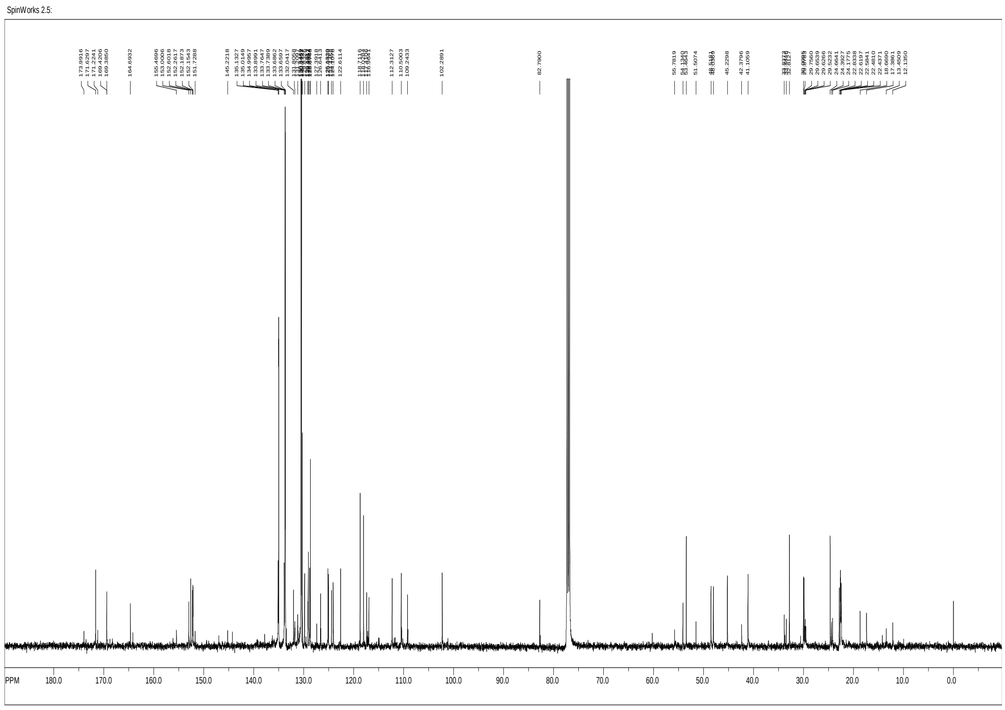

Fig. S11 ^{13}C NMR spectrum (125 MHz) of **TA1** in $CDCl_3$

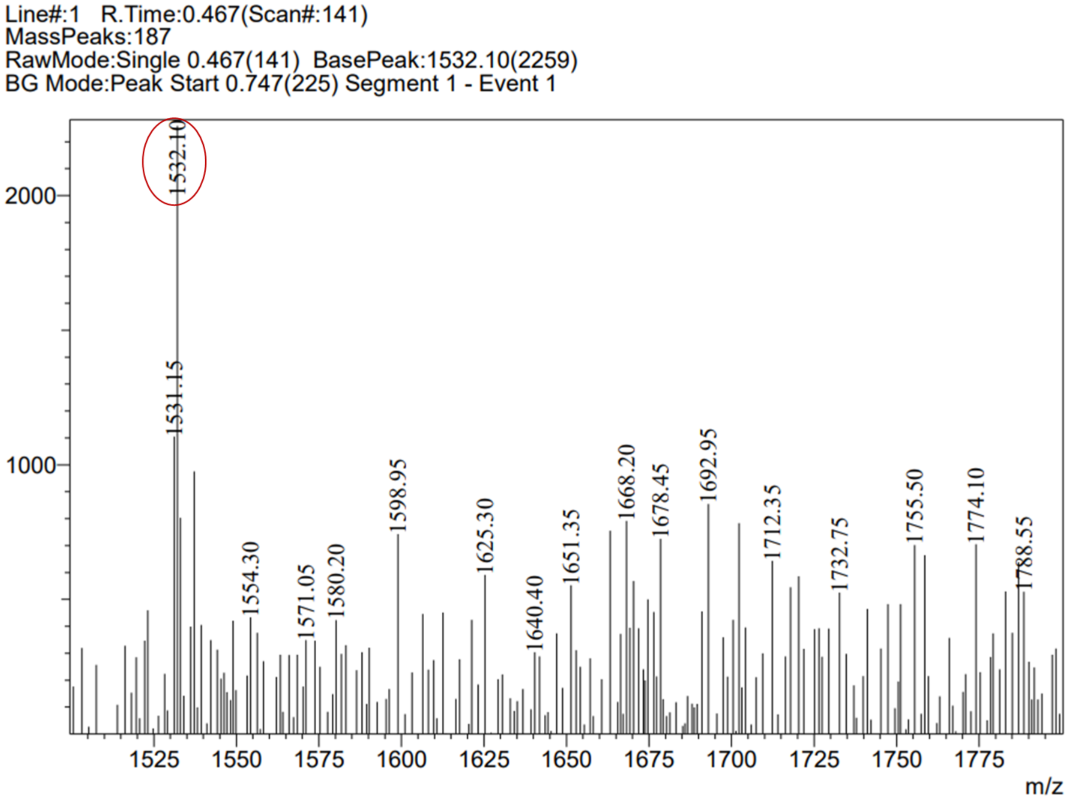

Fig. S12 ESI-MS spectrum of **TA1**

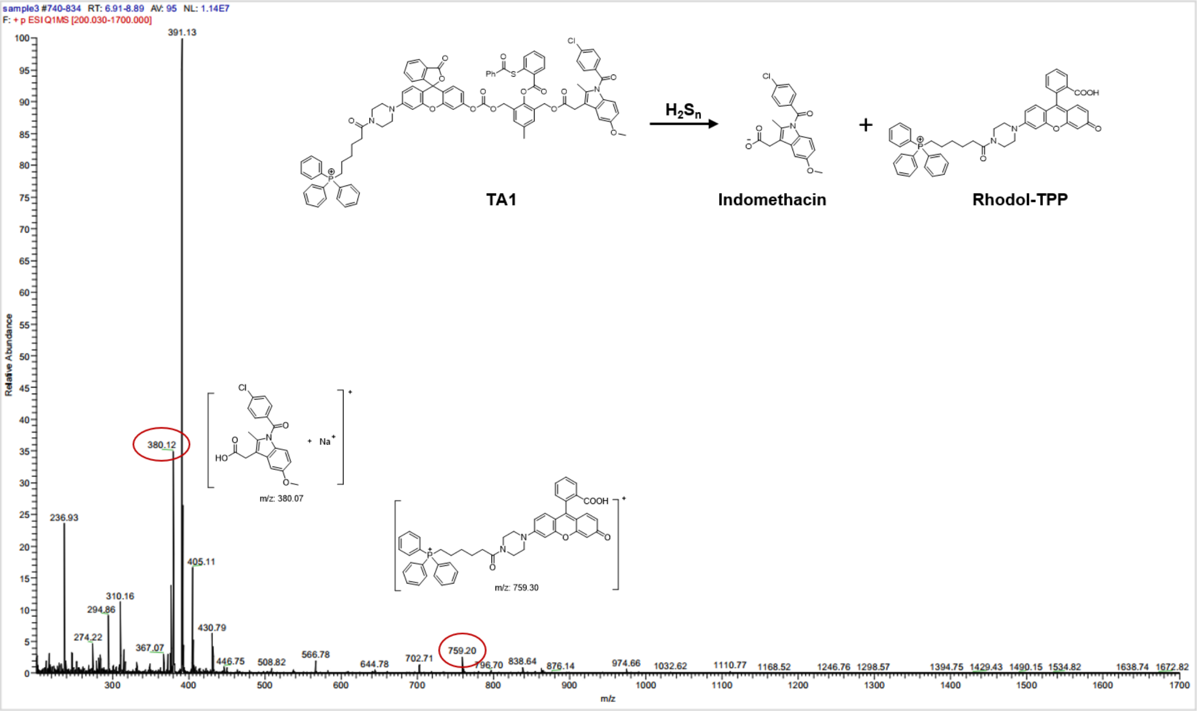

Fig. S13 ESI-MS spectrum of **TA1** after incubation with Na_2S_3 for 7 h in 10 mM PBS (pH 7.4)

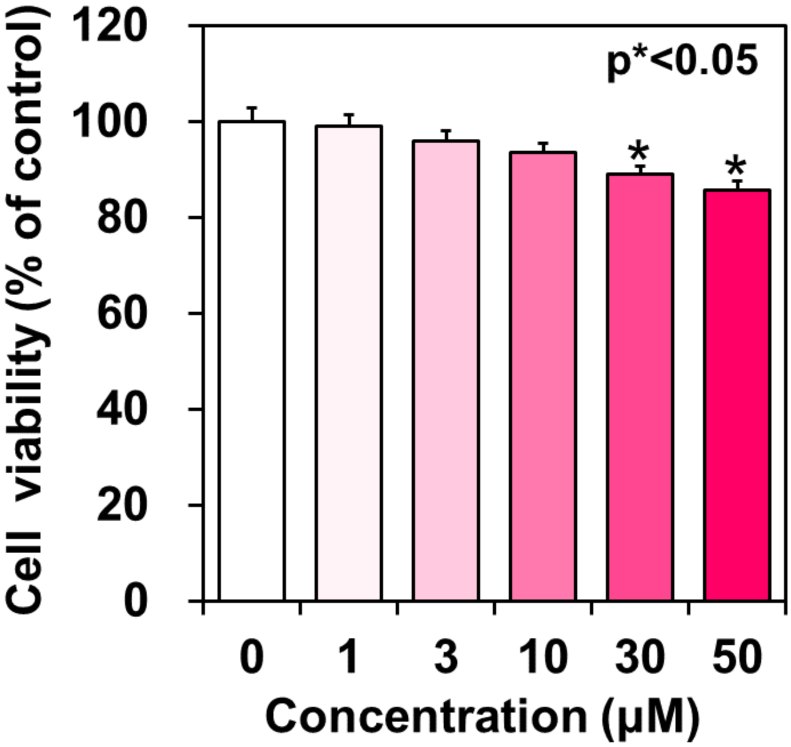

Fig. S14 Cell viability assay in RAW264.7 cell line determined by LDH assay upon various concentrations of **TA1**. It was measured after 24 h with the treatment of **TA1**

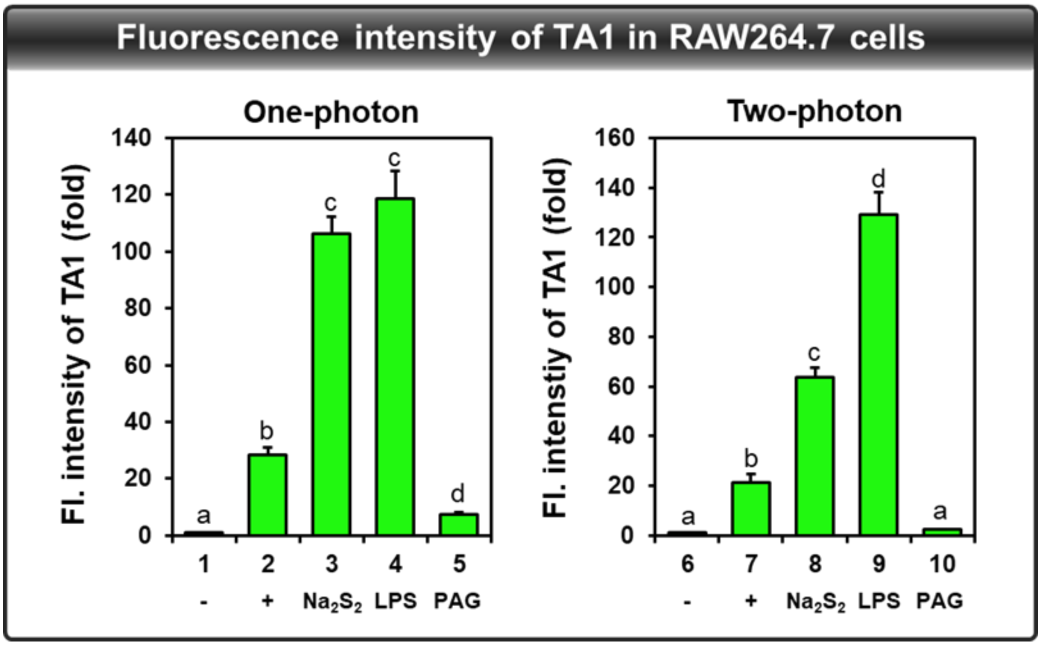

Fig. S15 Fluorescence intensities graph of **TA1** in Raw264.7 cells. This figure related to **Fig. 3**. **1** Control (1% DMSO), **2 TA1**, **3 TA1** + Na_2S_2 (5 μM), **4 TA1** + LPS (1 μg/mL), **5 TA1** + PAG (1 mM). Fluorescence images of cells were collected at λ_{ex} = 488 nm, λ_{em} = 500-560 nm of **TA1** and at λ_{ex} = 579 nm, λ_{em} = 580-620 nm of Mito-tracker. Two-photon fluorescence microscopy images in RAW264.7 cells incubated with **TA1** (10 μM) for 2 h. The cells in each group were subjected to different treatments. **6** Control (1% DMSO), **7 TA1**, **8 TA1** + Na_2S_2 (5 μM), **9 TA1** + LPS (1 μg/mL), **10 TA1** + PAG (1 mM). Statistical significance was determined by a one-way ANOVA test with a post-hoc Bonferroni test. Different letters (*e.g.*, a–d) signify datasets that are statistically distinct ($p < 0.05$).

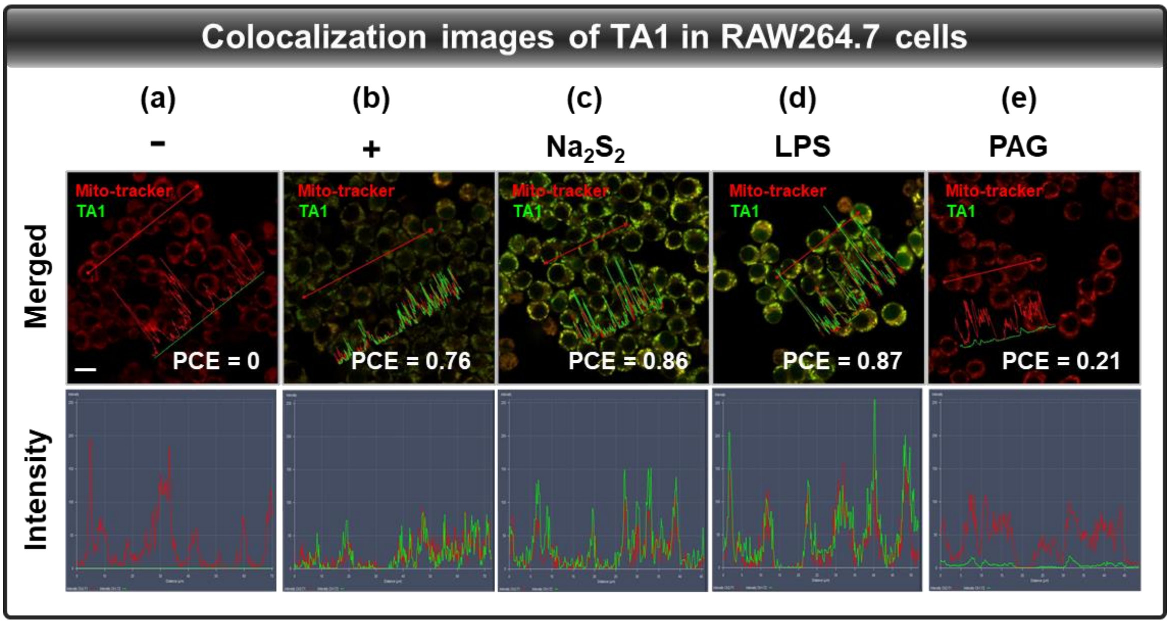

Fig. S16 *In vitro* test of **TA1** for colocalization cell imaging. Merged (**TA1** and Mito-tracker) and intensity graph images of colocalization in RAW264.7 cells incubated with **TA1** (10 μM) for 2 h and then stained using Mito-tracker for 30 min. The cells in each group were subjected

to different treatments. **a** Control (1% DMSO), **b TA1**, **c TA1** + Na_2S_2 (5 μM), **d TA1** + LPS (1 μg/mL), **e TA1** + PAG (1 mM). It was measured after 2.5 h with the treatment of **TA1** and sub-cellular tracker (Mito-tracker). Fluorescence images of cells were collected at λ_{ex} = 488 nm, λ_{em} = 500-560 nm of **TA1** and at λ_{ex} = 579 nm, λ_{em} = 580-620 nm of Mito-tracker. Scale bar = 100 μm. PCC = Pearson correlation coefficient.

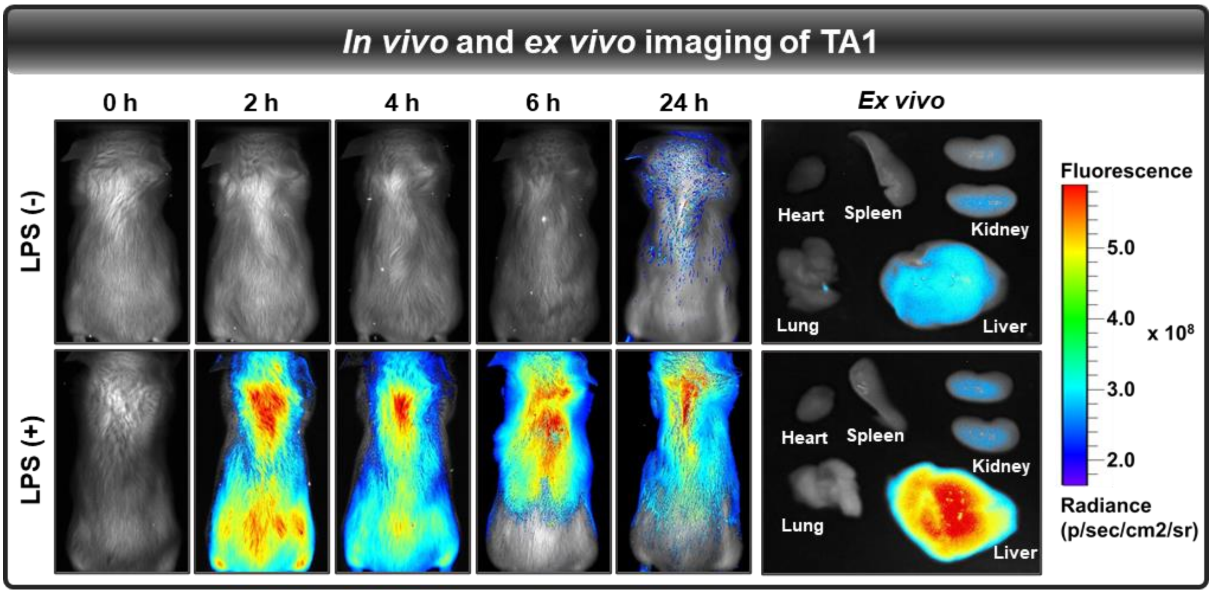

Fig. S17 *In vivo* and *ex vivo* imaging of **TA1** for drug distribution in mice. *In vivo* imaging of **TA1** at an indicated time point (0 h, 2 h, 4 h, 6 h, and 24 h) after 5% DMSO and LPS (*i.p.*) administration. *Ex vivo* imaging of **TA1** at 24 h after DMSO 5% and LPS (*i.p.*) administration.

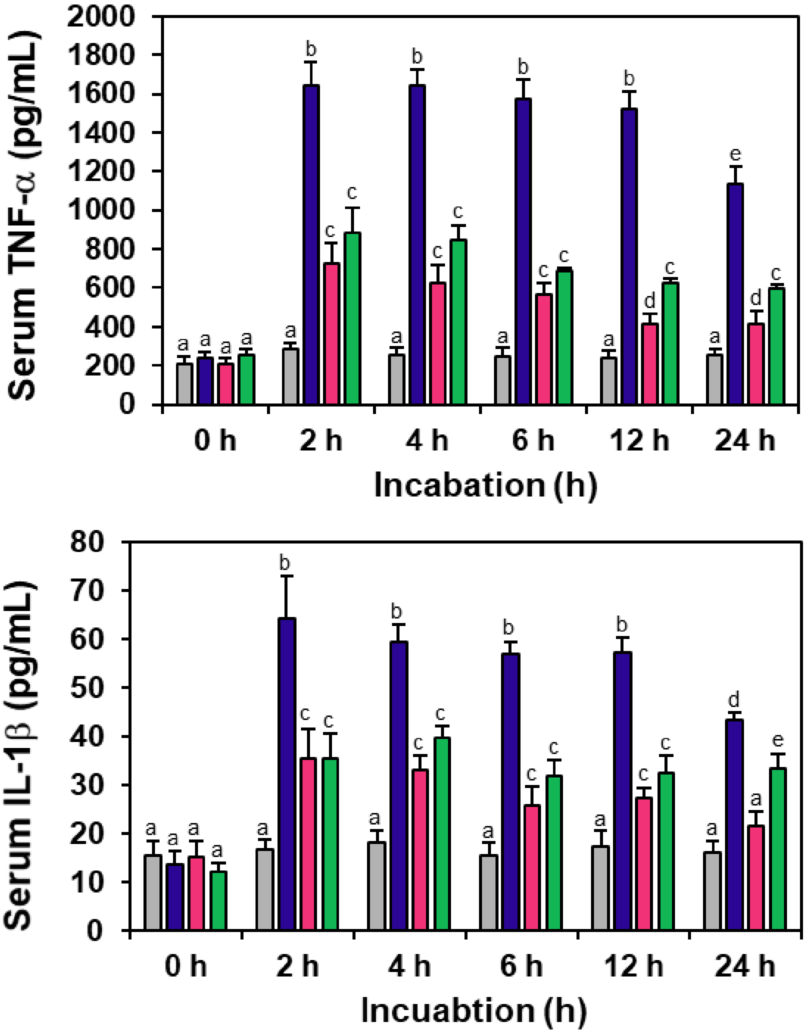

Fig. S18 Time-dependent-manners of serum TNF-α and IL-1β of LPS induced acute liver injury (ALI) mice. At indicated time points (0 h to 24 h), blood was collected and serum TNF-α and IL-1β levels were determined by ELISA. Results represent data from five mice. Control (5% DMSO); gray, ALI (LPS 10 μg/kg); deep blue, ALI + **TA1** (10 μg/kg LPS, 10 mg/kg **TA1**); pink and ALI + IMC (10 μg/kg LPS, 10 mg/kg IMC); green. Statistical significance was determined by a two-way ANOVA test with a post-hoc Bonferroni test. Different letters (*e. g.*, a–e) signify datasets that are statistically distinct ($p < 0.05$). ALI: LPS-induced acute liver injury, IMC: indomethacin

Supplementary Reference

[S1] S. Wu, S.Y. Tan, C.Y. Ang, Z. Luo, Y. Zhao, Oxidation-triggered aggregation of gold nanoparticles for naked-eye detection of hydrogen peroxide. Chem. Commun. **52**, 3508-3511 (2016). https://doi.org/10.1039/C5CC09447J
